# Hearing Function after CyberKnife for Vestibular Schwannoma: A Systematic Review *

**DOI:** 10.1055/s-0044-1787736

**Published:** 2024-07-05

**Authors:** Matheus Pedrosa Tavares, Fayez Bahmad Jr

**Affiliations:** 1 Postgraduate Program in Health Sciences, School of Medicine, Universidade de Brasília, Brasília, DF, Brazil

**Keywords:** vestibular schwannoma, radiosurgery, cyberKnife, hearing, systematic review, meta-analysis

## Abstract

**Introduction** CyberKnife (CK) radiosurgery is a treatment strategy for vestibular schwannoma (VS).

**Objectives** To evaluate hearing preservation (HP) after CK for VS.

**Data Synthesis** The study was conducted following the Preferred Reporting Items for Systematic reviews and Meta-Analyses (PRISMA) statement, and it was registered at the International Prospective Register of Systematic Reviews (PROSPERO, under number CRD42021250300). The inclusion criteria were based on the population, intervention, comparison, outcome, timing and study design (PICOTS) strategy: population – patients with VS; intervention – CK; Comparison – none; Outcome – serviceable HP defined by Gardner and Robertson as grades I or II, or by the American Academy of Otolaryngology and Head and Neck Surgery as classes A or B; timing – mean follow-up longer than 1 year; and study design – retrospective or prospective studies. The exclusion criteria were: studies not published in English; studies published before January 2000 and after October 2021; and studies only including patients with neurofibromatosis type 2 or submitted to a previous treatment. The PubMed/MEDLINE, EMBASE, Web of Science, Cochrane Library, LILACS, and IBECS databases were used and last searched on October 27th, 2021. Statistical heterogeneity was assessed using *I* ^2^ statistics. The appraisal checklist was used to assess the risk of bias in the included studies. A total of 222 studies were analyzed, and 13 were included in the synthesis, which represents 493 participants with serviceable hearing before intervention. The mean HP rate after CK using a random effects model was of 68% (95% confidence interval [95%CI]: 59–76%) at a mean follow-up of 42.96 months.

**Conclusion** The longer follow-up period was associated with a lower HP rate after CK radiosurgery for VS in the qualitative synthesis.

## Introduction

Vestibular schwannoma (VS), or acoustic neuroma, is a benign tumor of the vestibulocochlear nerve, the eighth cranial nerve, and its incidence has increased mainly due to widespread access to neurodiagnostic imaging tests. 1 2 3 The current incidence rates range from 3 to 5 cases per 100 thousand person-years. 1 3 Vestibular schwannoma is sporadic in most cases; however, there is an association to neurofibromatosis type 2 (NF2) in less than 5% of the cases, 4 which is a factor of worse prognosis and greater risk of developing bilateral disease. 5 The treatment options for VS are the wait-and-scan approach, radiotherapy, and microsurgery. The aims of the management are tumor control and symptom control. To decide which treatment strategy will be used, the morbidity of each patient needs to be considered. Some adverse events related to the treatment are dysfunctions in the vestibulocochlear nerve, facial nerve, trigeminal nerve, and lower cranial nerve, bleeding, cerebrospinal fluid leak, hydrocephalus, meningitis, and stroke.

Stereotactic radiosurgery (SRS) involves the use of radiation directed to the lesion of interest as a target. The aim is to prevent tumor expansion, in opposition to the aim of microsurgery, which is total or subtotal removal of the lesion in selected cases. Stereotactic radiosurgery attempts to attenuate the impact of radiation on the tissues surrounding the lesion and, consequently, reduce the morbidity related to nerve damage. The types of radiation used are gamma knife (GK) – the object of most studies about SRS and hearing preservation (HP), linear accelerator (LINAC), proton beam therapy, and CyberKnife (CK, Accuray, Sunnyvale, California, United States) a robotic frameless system of LINAC-based radiosurgery. 6

The treatment decision in VS is mostly custom-tailored to the individual situation of the patient. There is no high-quality evidence determining the superiority of any of the treatment options for VS. 7 Studies have been conducted to determine the effectiveness of the wait-and-scan approach, microsurgery, radiotherapy, and each radiation type based on tumor control, trigeminal and facial nerve function, and HP.

The association between the lower probability of HP and longer period of follow-up has been demonstrated in studies about radiosurgery techniques in general. 8 9 Although CK studies were included in these previously cited articles, there is no mention of this association specifically for CK in the literature. The study by Mahboubi et al. 10 (2017) is the only systematic review that includes CK studies individually, and it only describes the mean HP rate. Moreover, there are no studies thoroughly investigating the hearing aspects after CK in patients with VS. Personal observation of hearing deterioration after CK radiosurgery in our department had spurred the present investigation.

The objectives of the present study are to determine the HP rate after CK according to the mean follow-up period of the included studies, and to evaluate the association between the probability of HP and the duration of the follow-up, as well as other variables.

## Review of the Literature

The present study was conducted in accordance with the guidelines of the Preferred Reporting Items for Systematic Reviews and Meta-Analyses (PRISMA) statement. 11 The study protocol was registered at the International Prospective Register of Systematic Reviews (PROSPERO, under number CRD42021250300).

### Search Strategy

To identify studies for inclusion, a systematic search of the literature was performed in the following databases: PubMed/MEDLINE, Excerpta Medica database (EMBASE), Web of Science, Cochrane Library, Latin American and Caribbean Health Sciences Literature (LILACS), and Spanish Bibliographic Index on Health Sciences (IBECS). All databases were last searched on October 27th, 2021. No automation tools, filters or limits were used in the search. The search strategy used in all databases was: ( *vestibular schwannoma* OR *acoustic neuroma* ) AND ( *cyberknife* ). A manual search was performed in reference lists from other studies.

After the search, the results of each database were exported to the Zotero (open source; https://www.zotero.org ) and Rayyan (Rayyan Systems Inc., Cambridge, MA, United States; https://www.rayyan.ai ) software. The purpose of using these two software was to increase the reliability of the selection of articles and the identification of duplicate studies before the eligibility stage.

### Eligibility Criteria

The review question applied to the present study was: “What is the HP rate after CK radiosurgery in patients with VS?”. The population, intervention, comparison, outcome, timing, and study design (PICOTS) strategy was used to define eligibility criteria in accordance with the review question. The inclusion criteria were: population – patients with VS; intervention – CK radiosurgery; comparison – none; outcome – serviceable or useful HP defined by the hearing classification systems by Gardner and Robertson (GR) 12 as grades I or II, and by American Academy of Otolaryngology and Head and Neck Surgery (AAO-HNS) 13 as classes A or B ( Table 1 ), or pure tone average (PTA) ≤ 50 dB, or speech discrimination score (SDS) ≥ 50%; timing – mean follow-up longer than 1 year; and study design – clinical trials, cohort and case-control studies, case series, retrospective or prospective studies.

**Table 1:** Definition of serviceable hearing according to the Gardner-Robertson (I–II) 12 and AAO-HNS (A–B) 13 hearing classification systems

Grade/Class	Hearing level	PTA (dB)	SDS (%)
I/A	Good	0–30	70–100
II/B	Serviceable	31–50	50–69

**Abbreviations:** AAO-HNS, American Academy of Otolaryngology and Head and Neck Surgery; PTA, pure tone average; SDS, speech discrimination score.

The exclusion criteria were: papers not published in English; studies published before January 2000 and after October 2021, because the treatment protocols prior to 2000 are different from the modern dosages; incomplete studies (such as conference abstracts); studies using animals and in-vitro studies; case reports; studies only including patients with NF2 or those submitted to a previous treatment (such as radiotherapy or microsurgery); use of a source of radiation other than CK (such as GK, LINAC, proton beam therapy); inadequate data report regarding the number of patients with serviceable hearing pre- and posttreatment or the documented time of follow-up; and repeated data from a previous study.

We initially planned to exclude studies with NF2 patients and those submitted to a previous treatment, because these two variables are potential factors for a worse probability of HP. Since almost all studies included patients with at least one of the aforementioned conditions and most of the patients with these conditions would not present serviceable hearing before CK, we decided to not use it as exclusion criteria.

The following stages of the study were performed independently by the same two blinded reviewers. The decisions of each reviewer were recorded in separate documents. Any disagreements between them were solved through a discussion.

### Study Selection

The study selection was conducted in two phases. In the first stage, after the duplicates were removed, the studies were identified by title and abstract analysis. In the next stage, a full-text analysis of the screened studies was performed. The studies that met the eligibility criteria were included in the qualitative analyses.

### Data Extraction

The following data were extracted individually from each study: name of the authors; year of publication; location (country); prospective or retrospective design; total number of patients; radiation dosage regimen; hearing classification system used to define serviceable hearing; number of patients with serviceable hearing pre- and posttreatment; HP rate in percentage; number of patients with NF2 and submitted to a previous treatment – radiotherapy or microsurgery; duration of the follow-up in months or years, preferably the mean value instead of the median, and representing the audiometric follow-up, or the general follow-up of the study, if the first one was not reported; and variables associated with HP.

### Methodological Quality Assessment

An analysis of the risk of bias in the included studies was performed independently by two blinded reviewers according to the appraisal checklist for case series studies of the Joanna Briggs Institute (JBI; https://jbi.global/critical-appraisal-tools ). In this checklist, each of the ten questions about the study's methodology must be answered through four options: yes (Y), no (N), unclear (U), or not applicable (NA). The risk of bias is calculated by the number of Y answers, and it is classified as high (≤ 49%), moderate (50–70%) or low (≥ 71%);. the NA answers are not considered in the calculation. The directness of the evidence was evaluated considering if each study design matched the PICOTS strategy used in the present study.

### Statistical Analysis

The quantitative analysis was conducted with data from the included studies, and it used the number of patients with serviceable hearing before and after CK. To calculate the mean HP rate, the decision on whether to use a fixed or random effects model was based on the statistical heterogeneity of the studies, which was assessed using *I* ^2^ statistics. The interpretation of the *I* ^2^ statistic was based on the following modified thresholds from Higgins et al. 14 (2003): < 25% – no considerable heterogeneity; ≥ 25% – low; ≥ 50% – moderate; and ≥ 75% – high. As also recommended by Higgins et al., 14 in the qualitative analysis of heterogeneity, diversity in the following clinical and methodological aspects of each study was considered in the evaluation: treatment protocol; clinical characteristics of the participants, regarding the number of NF2 patients and of those submitted to a previous treatment; and the follow-up period. In case there was high or moderate statistical heterogeneity and considerable diversity in the clinical and methodological aspects, a random effects model would be used in meta-analysis, and in the case of low heterogeneity, a fixed effect model would be used. Statistical significance was set at *p* < 0.05. All statistical tests were performed using the Jeffreys's Amazing Statistics Program (JASP, open source), version 0.14.1, and the “meta” package from the R software (R Foundation for Statistical Computing, Vienna, Austria), version 4.0.5.

## Results

### Study Selection

**Fig. 1:**
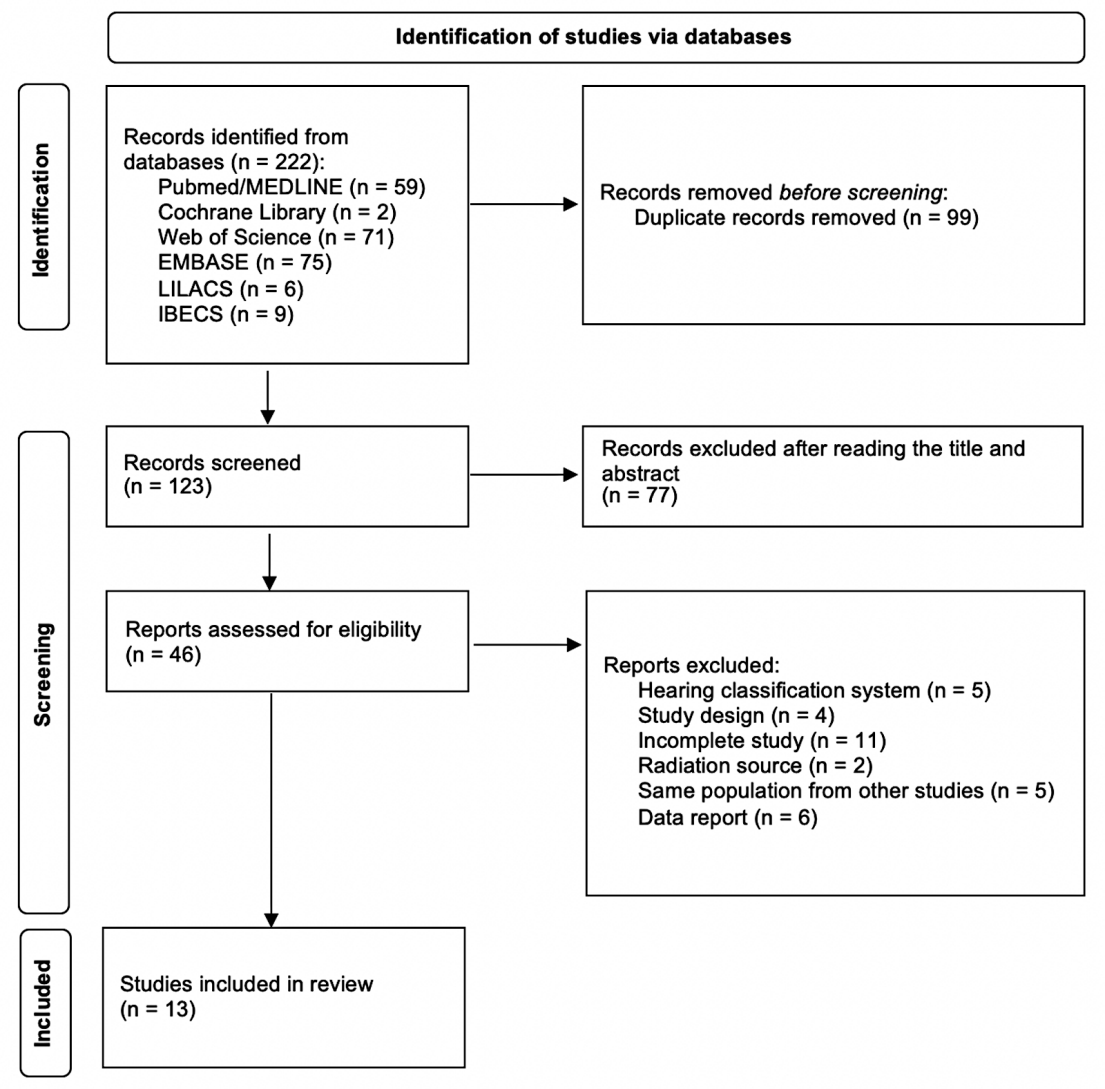
Flow diagram of study selection modified from the Preferred Reporting Items for Systematic Reviews and Meta-Analyses (PRISMA) statement.

A summary of the selection of studies is shown in Fig. 1 . The search in 6 databases yielded a total of 222 studies, and no studies were selected from reference lists. After 99 duplicates were removed, 123 articles were submitted an analysis of the title and abstract, and 77 were excluded. The 46 remaining articles had their full text read, and 33 were excluded due to the following reasons: the hearing classification system specified in the eligibility criteria was not used or it was used inadequately (for example: considering GR grade III or AAO-HNS class C as serviceable hearing); 15 16 17 18 19 inadequate study design; 20 21 22 23 incomplete study; 24 25 26 27 28 29 30 31 32 33 34 use of another radiation source; 35 36 same population from others studies; 37 38 39 40 41 and inadequate data report 42 43 44 45 46 47 (the corresponding authors of these studies were contacted to request additional information, but there was no response). Then, a total of 13 studies 48 49 50 51 52 53 54 55 56 57 58 59 60 were included in the analysis.

### Study Characteristics

The data extracted from each study is presented in Table 2 . In general, regarding the study design, 11 (85%) articles were retrospective, and 2 (15%) were prospective case series. About the hearing classification system used to define serviceable hearing, the GR was used in 8 (61%) studies, the AAO-HNS, in 3 (23%), the AAO-HNS and GR, in 1, and PTA ≤ 50 dB, in 1 study. The mean radiation dosage ranged from 17 to 24.78 Gy, and it was delivered in a fractionated regimen in nearly all of the cases. The mean number of sessions in studies ranged from 2.5 to 4.89. The exact dosage regimen of each study is described in Table 3 .

**Table 2:** Summary of the characteristics of the included studies

Study	Country	Design	Total sample	no. NF2 (%)*	no. previous treatment (%)*	Definition of serviceable hearing	Serviceable hearing sample	Hearing preservation rate (%)	Follow-up in months (range)
Ishihara et al. (2004) 48	Japan	Prosp	38	0 (0)	11 (29)	GR I-II	14	92.86	31.9 (12–59); mean
Hansasuta et al. (2011) 49	USA	Retro	383	15 (4)	41 (11)	GR I-II	200	75.50	36 (12–106.8); median
Karam et al. (2013) 50	USA	Retro	37	0 (0)	0 (0)	GR I-II	14	78.57	18 (NR); median
Lin et al. (2013) 51	Taiwan	Prosp	20	0 (0)	0 (0)	GR I-II and AAO-HNS A-B	10	50.00	24 (NR); mean
Morimoto et al. (2013) 52	Japan	Retro	25	5 (20)	6 (24)	PTA ≤ 50 dB	12	50.00	101 (3–161); median
Tsai et al. (2013) 53	Taiwan	Retro	117	0 (0)	24 (21)	GR I-II	65	81.54	64.5 (21–89); mean
Vivas et al. (2014) 54	USA	Retro	73	NR	10 (14)	AAO-HNS A-B	28	53.57	36 (NR); mean
Casentini et al. (2015) 55	Italy	Retro	33	2 (6)	0 (0)	GR I-II	8	87.50	48 (12–111); median
Çakır et al. (2018) 56	Turkey	Retro	26	NR	0 (0)	GR I-II	16	68.75	16.4 (12–30); mean
Gallogly et al. (2018) 57	USA	Retro	22	NR	NR	AAO-HNS A-B	11	36.36	36.7 (NR); mean
Przybylowski et al. (2019) 58	USA	Retro	119	4 (3)	25 (21)	AAO-HNS A-B	59	59.32	49 (6–133); median
Pialat et al. (2021) 59	France	Retro	82	0 (0)	16 (20	GR I-II	28	46.43	24 (6–81); median
Puatapeewong et al. (2021) 60	Thailand	Retro	123	NR	NR	GR I-II	28	78.57	72 (12–123); median

**Abbreviations:** AAO-HNS, American Academy of Otolaryngology and Head and Neck Surgery classification; GR, Gardner-Robertson classification; NF2, neurofibromatosis type 2; NR, not reported; Prosp, prospective; PTA, pure tone average; Retro, retrospective

**Note:** *Percentage of the total sample.

**Table 3:** Radiation dosage and cochlear doses

Study	Mean radiation dosage (Gy)	Mean number of sessions	Cochlear dose (Gy)	
			Maximum	Minimum
Ishihara et al. (2004) 48	17	2.5	NR	NR
Hansasuta et al. (2011) 49	18	3	NR	NR
Karam et al. (2013) 50	24.78	4.89	NR	NR
Lin et al. (2013) 51	18	3	NR	NR
Morimoto et al. (2013) 52	21	3	NR	NR
Tsai et al. (2013) 53	18	3	Group with worse hearing: 15.257; preserved hearing: 12.07 (mean)	Group with worse hearing: 0.92; preserved hearing: 0.65 (mean)
Vivas et al. (2014) 54	18	3	Posttreatment GR A: 15.2; GR B: 16.4 (mean)	Posttreatment GR A: 1.9; GR B: 2.8 (mean)
Casentini et al. (2015) 55	17.5	3.36	NR	NR
Çakır et al. (2018) 56	19.08	4	NR	NR
Gallogly et al. (2018) 57	21	3	NR	NR
Przybylowski et al. (2019) 58	18	3	18 (median)	NR
Pialat et al. (2021) 59	22.41	3.7	NR	NR
Puatapeewong et al. (2021) 60	18.96	3.32	18-Gy group: 45.2; 20-Gy group: 53.3; 25-Gy group: 61.9* (median)	18-Gy group: 10.7; 20-Gy group: 14.5; 25-Gy group: 32.8* (median)

**Abbreviations:** GR, Gardner-Robertson classification; NR, not reported.

**Note:** *Biological effective dose (BED)

### Quantitative Synthesis

All 13 studies included represent a total of 493 patients with serviceable hearing before and 346 after CK. The crude mean HP rate was of 70.18% (standard deviation [SD]: ± 12.64), and it ranged from 36.36 to 92.86%. The weighted mean follow-up was of 42.96 months (SD: ± 17 months; 3.58 years) and it ranged from 16.4 to 101 months (1.36 to 8.41 years).

**Fig. 2:**
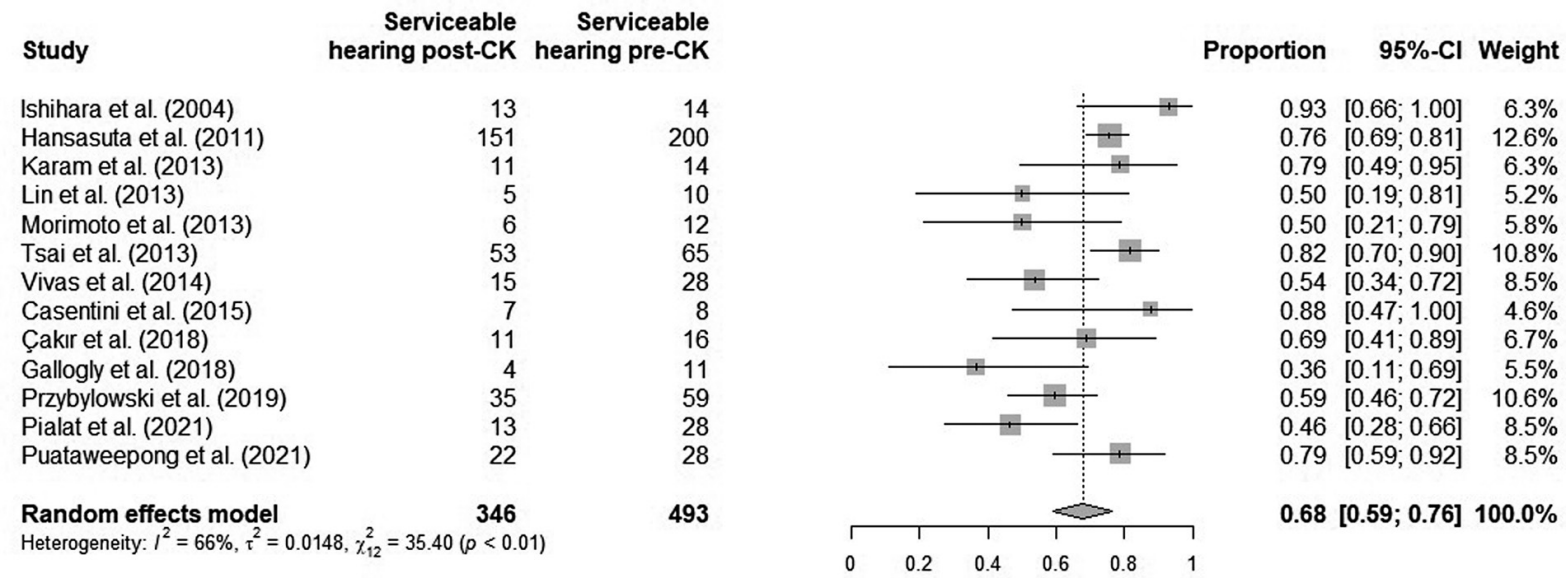
Forest plot of the included studies and each hearing preservation rate.

The statistical heterogeneity among the included studies was moderate, of 66% ( *p* < 0.01). Diversity in the clinical and methodological aspects was identified, especially regarding the percentages of NF2 patients and those submitted to a previous treatment, and the follow-up range, which were different in many studies and were not reported in some of them. Therefore, we decided to use a random effects model to determine the aggregate HP rate, which was of 68% (95% confidence interval [95%CI]: 59–76%). A forest plot of the included studies is presented in Fig. 2 .

## Qualitative Synthesis

### Duration of the Follow-up

In Gallogly et al. 57 (2018), the HP rate calculated through the Kaplan-Meier method was of 51.1% at 3 years of follow-up, and of 17.5% at 5 years. In Pialat et al. 59 (2021), the HP rate was of 75.6% at 1 year and of 64.3% at 2 years; the mean time until hearing degradation was of 29.4 (95%CI: 23.5–35) months. In Puataweepong et al. 60 (2021), the HP rates after 5 and 8 years were of 87% and 65% respectively, and the median time until hearing deterioration was of 71 (range: 24–92) months. Morimoto et al. (2013) 52 reported progressive deterioration of the PTA in 92% of patients; the ean PTA levels before and after CK were of 29.8 and 57.1 dB respectively, and statistical differences were not reported. Lin et al. (2013) 51 reported mean PTA levels before and after CK of 55 and 66 dB respectively, with no statistically significant difference ( *p* > 0.05).

### Age

In Hansasuta et al. 49 (2011) and Çakır et al. 56 (2018), the age of the patients was not associated with the probability of HP ( *p* = 0.692 and 0.06 respectively), although in the former, younger age tended to be associated with better HP.

### Tumor Volume

Çakır et al. 56 described no association between tumor size and the probability of HP ( *p* = 0.532). In Hansasuta et al., 49 a smaller tumor volume (as a continuous variable) was associated with higher HP rate ( *p* = 0.001), specifically tumors smaller than 3 cm ^3^ ( *p* = 0.009). In Tsai et al. 53 (2013), larger tumor sizes were associated with a worse probability of HP ( *p* < 0.001)

### Radiation Dosage

Çakır et al. 56 described no correlation between radiation dosage and the probability of HP ( *p* = 0.286), and Tsai et al. 53 described an association between higher doses and a lower probability ( *p* < 0.001).

### Koos Grade 61

In Hansasuta et al., 49 the HP rate of Koos grades II, III and IV tumors (73%) were significantly lower than that of Koos grade I (83%; *p* = 0.019). Karam et al. 50 (2013) reported an HP rate of 100% in patients with Koos grade I, and of 72% in those with Koos grades II, III and IV ( *p* -value not reported).

### Cochlear Volume

Tsai et al. 53 reported an association between smaller cochlear volume and worse probability of HP ( *p* < 0.001). Pialat et al. 59 described no association between cochlear volume and HP. No statistical difference was reported.

### Pre-CK Hearing Grade or Class

Tsai et al. 53 described that 92% of the patients who experienced hearing deterioration from serviceable to unserviceable hearing were GR grade II before CK. In Vivas et al. 54 (2014), HP was obtained in 77% of the patients who had class A hearing pre-CK class, and in 33% of thosde who had class B hearing pre-CK according to the AAO-HNS classification. No statistical difference was reported. Çakır et al. 56 reported HP in 85.7% of the patients with a pre-CK PTA ≤ 20 dB and in 70% of those with a pre-CK SDS ≥ 80%.

### Risk of Bias and Quality of Evidence Assessment

In summary, a low risk of bias was found in all studies. Each included study was analyzed according to the JBI appraisal checklist for case series. The design of all studies matched the PICOTS strategy. Consequently, the directness of evidence was high.

## Discussion

A synthesis of the results from 13 studies showed an HP rate of 68% at 43 months of follow-up after CK radiosurgery for VS using a random effects model. Mahboubi et al. 10 (2017) performed a systematic review specifically for CK studies: The HP rate was of 79.1%, and there was no report of mean time of follow-up. Yang et al. 62 (2010) conducted a meta-analysis of GK studies, and the HP rate was of 51% at a mean follow-up of 44.4 months. Fong et al. 63 (2012), in a meta-analysis of LINAC studies, found mean HP rates of 66.3% at mean follow-up of 45 months for SRS studies and of 75.3% at 38.5 months for fractionated SRS studies. Coughlin et al. 8 (2018) included papers about LINAC and GK radiosurgery in another systematic review. The HP rate was of 58% at a mean follow-up of 46.6 months.

Coughlin et al. 8 also described HP rates of 73% at less than 2 years of follow-up of 60% between 2 and 5 years, of 48% between 5 and 10 years, and of 23% at more than 10 years. There was statistically significant difference among these results ( *p* = 0.00001). Carlson et al. 9 (2018) reported in a guideline that 72% of patients submitted to radiosurgery maintained serviceable hearing at 2 years of follow-up, 63%, at 5 years, and 33%, at 10 years. Basically, these studies showed a progressive deterioration of HP as the follow-up increases.

In the qualitative synthesis, five studies 51 52 57 59 60 showed progressive hearing degradation. Most of the evaluated variables (patient age, tumor volume, radiation dosage, and cochlear volume) were not associated with HP, or there were conflicting results among studies. The Koos grade and pre-CK hearing grade or class were the only variables associated with hearing in the qualitative synthesis: Koos stage-I tumors, in two studies, 49 50 and patients with pre-CK grade I or class A, in three studies, 53 54 56 exhibited better HP rates. The latter finding was also observed by Carlson et al. 9 who reported the association of the following factors with serviceable HP after radiosurgery: good preintervention SDS scores or PTA levels; smaller tumor volume; marginal tumor dose ≤ 12 Gy; and cochlear dose ≤ 4 Gy. Yang 62 reported superior HP rates with a lower radiation dose (≤ 13 Gy; *p* < 0.0005). In Fong, 63 larger tumors (≥ 3 cm ^3^ ) presented better HP rates compared with smaller tumors when submitted to fractionated stereotactic radiotherapy ( *p* = 0.004).

## Limitations

A meta-analysis to investigate the association between the probability of HP and the duration of the follow-up could not be performed due to the inappropriateness of the data. The main reasons were heterogeneity in the included studies, inconsistent hearing reports, and loss to follow-up bias, as the sample of studies is presumably smaller in the longer time points of the follow-up period.

There are some points that may compromise the quality of evidence of the present study: 1) in some of the included articles, the general time of follow-up for the whole sample was reported, but there was no description of the follow-up period for audiological tests or mention of the serviceable hearing sample specifically; 2) all included studies were nonrandomized studies, with no control groups; and 3) the design of most of the included studies was retrospective.

The heterogeneity among the studies was quite high due to the aforementioned points, and particularly because the range of HP rates and follow-up periods among studies were considerably wide. In each study, different proportions of patients with grade I/class A and grade II/class B hearing, or with Koos stage I and other stages may be the reason for the great differences in HP rates, as these variables were associated with better hearing prognosis. Besides, the broad spectrum of follow-up periods in each study raises the question about the possibility of attrition and reporting biases, and, consequently, about the validity of certain findings. The conflict of interests in the studies should also be considered a source of bias.

## Future Studies

Multi-institutional, randomized controlled trials with a prospective design are required to better evaluate the role of CK in hearing degradation after radiosurgery. Standardized reporting of results in raw audiometric data are needed to improve our understanding of hearing outcomes attributable to CK and to describe HP in more detail. Further studies should also aim to have longer follow-up periods, to evaluate how much the hearing deterioration after CK is influenced by time. The mean or median values of the analyzed variables were relative to the total sample, not specific to the serviceable hearing sample. Therefore, comparisons involving these variables and the probability of HP in a meta-analysis could not be performed. Studies focusing on patients with serviceable hearing before CK and reporting these numbers adequately may provide the means to conduct meta-analyses in the future. Additionally, it would contribute to determine, with consistent results, which factors are associated with the probability of HP.

## Final Comments

Longer follow-up, worse pre-CK hearing grade or class, and Koos grade II or higher were associated to lower HP rates after CK radiosurgery for VS in a qualitative synthesis.
